# Clinical Case

**Published:** 1885-02

**Authors:** R. B. Johnstone

**Affiliations:** Pittsford, N. Y.


### CLINICAL BUREAU.

### CLINICAL CASE.

Dr. R. B. Johnstone, Pittsford, N. Y.

Miss M. K., aged forty-three; blue eyes, auburn hair, fair skin and freckles, lips rather thick and pouting; occupation, teaching and bookkeeping, in which business she had been engaged for some years, when I was called to see her on June 22d, 1881.

Found her in profound prostration and great nervousness, Could not remain in bed, but must get up and change her position often. Cannot walk, but she is inclined to fall one side or the other.

Memory weak; thinks with difficulty; unable to actively engage her mind; she is so tired after a little thinking.

Headache mostly in the left side. Dullness of sight; sees only part of an object, sometimes one half, at others the other half.

Prickling in the eyes when reading or looking at the light.

Very sensitive to odors of all kinds, which are very disagreeable to her; even pleasant odors of hay, clover, in which she used to delight, now literally "stink."

Appetite is gone; have no desire to eat; everything tastes like "old rags."

Stomach is sour and feels hot; has been in the habit of taking "soda" for this trouble, which relieves her.

Constipation. The desire for stool is great, but have not the power to expel it, only wind or a little slime passes. Urine is filled with a red, sandy sediment, while the top of the urine looks as though it was covered by a drop of coal oil; the urine has a putrid smell.

Walking makes her tired and out of breath; inclined to sudden anger without cause, followed by gasping and short breath; this passes off after exertion of will. At times it seems as though my heart would jump out of my mouth.

My feet are constantly cold, also my head, while my ears are hot and very red.

When a child was always sickly, and had all children's diseases, but always got well quickly. Had swellings of the glands of the neck years ago; some of them opened.

About two months ago discovered a lump in the right side of abdomen, accompanied by sense of weight and dragging pain.

Menstruation too late and very scanty—often five or six days behind time, and last only a day or two.

Leucorrhœa is yellow greenish and excoriating.

On examination the tumor in the abdomen proved to be within the right ovary; it is hard, about the size of an orange, or three inches in diameter, without pain. The uterus is enlarged and cervix indurated.

℞. Sepia 200 D, in water, to be given every three hours till morning.

June 23d.—About the same. Rested easier. ℞. Sach. lac.

June 25th.—Still improving. Continued the "s. l."

June 29th.—About the house. Most of the "bad" symptoms have gone, but the "lump" in the abdomen seems twice the size it was two weeks ago, it is so heavy. Sleeps well. Appetite is growing better. ℞. Sach. lac.

July 21st.—Called at the office. Am very weak, but improving. Am sure the tumor is growing at a rapid rate. Have a diarrhœa which begins about 3 A. M., and continues until noon. Eyes ache; can hardly see; itching of the lids. My feet and hands are very cold; cannot get them warm.

Melancholy. Hypochondrical; cannot think.

℞. Sulph. 81M, F., two doses, followed by "s. l."

September 10th.—Reports continued improvement since the first dose in every respect but the tumor, which continues to grow at an alarming rate; cannot now get my skirts together.

Gave at various time "s. l." until October 18th, when in consultation with Dr. Biegler, of Rochester, after much trouble and questioning, learned that when she is alone she becomes very *melancholic, with serious thoughts of suicide,* which she tries to banish.

An examination per vag. and rectum disclosed the tumor, hard and rather uneven surface, taking up the whole right ovary, or more than four times its size, when first discovered, and after only five months' growth. No pain, except drawing, dragging pains in right abdomen and thigh; feet are so cold they really seem as though they "smarted;" eyes protrude; drawing in the eyes. Things look mixed up, or sees only one-half of the body looked at, usually the left half; other half covered as by a dense cloud.

Still sensitive to odors; menses late and scanty.

White or yellowish leucorrhœa, with burning and smarting.

Dyspnœa; constant desire to take a long breath.

Remembers now that when a child she had trouble in breathing, accompanied with swelling of the cervical glands; general scrofulous swellings in various parts of the body, but had nothing of the kind since old Dr. —— gave me what he called "*gold pills*."

I took a great many of them, also other medicines, which I think were what he called "blood purifiers."

This last decided beyond all doubt the proper remedy; so, on that day she received one dose of Aurum met.$^{200}$. Some two weeks after, Aurum met.$^{10\,m}$ was given, one dose, followed by sach. lac. Saw her frequently from that time on, but she received no more medicine of any kind.

January 10th, 1883.—Made another examination; found the tumor reduced in size to less than when first discovered; no pain, no dragging or pressure—menses regular and natural in quantity, color, and time; mental condition all cleared up; never thinks of the tumor now, unless asked about it. Gave one dose of Aurum$^{cm}$.

August, 1884.—"The tumor continues to grow less and gives no trouble; mind clear and have no trouble with the eyes, and have had none for a year or more; discharge from the right ear offensive and swashing in the head; feet get cold once in a while, with hot, red face at times; appetite good; stool passes each day without trouble; urine seems to be in a healthy condition. Indeed, I have no trouble now but the discharge from the ear."

Sulph.$^{cm}$, Swan, one dose.

Once since the above, was called upon again to prescribe for the ear, but thought best to wait for a while before giving another remedy.

Query. Did I cure a fibroid of the ovary or not?

Was it caused by the "gold pills" taken years ago?

Do high potencies contain any medicine? Do they act, or not?

